# An Iterative Approach for the Optimization of Pavement Maintenance Management at the Network Level

**DOI:** 10.1155/2014/524329

**Published:** 2014-03-11

**Authors:** Cristina Torres-Machí, Alondra Chamorro, Carlos Videla, Eugenio Pellicer, Víctor Yepes

**Affiliations:** ^1^Universitat Politècnica de València, Camino de Vera, s/n, 46022 Valencia, Spain; ^2^Escuela de Ingeniería, Departamento de Ingeniería y Gestión de la Construcción, Pontificia Universidad Católica de Chile, Avenida Vicuña Mackenna 4860, Edificio San Agustín, Piso 3, 7820436 Santiago, Chile; ^3^ICITECH, Department of Construction Engineering, Universitat Politècnica de València, Camino de Vera, s/n, 46022 Valencia, Spain

## Abstract

Pavement maintenance is one of the major issues of public agencies. Insufficient investment or inefficient maintenance strategies lead to high economic expenses in the long term. Under budgetary restrictions, the optimal allocation of resources becomes a crucial aspect. Two traditional approaches (sequential and holistic) and four classes of optimization methods (selection based on ranking, mathematical optimization, near optimization, and other methods) have been applied to solve this problem. They vary in the number of alternatives considered and how the selection process is performed. Therefore, a previous understanding of the problem is mandatory to identify the most suitable approach and method for a particular network. This study aims to assist highway agencies, researchers, and practitioners on when and how to apply available methods based on a comparative analysis of the current state of the practice. Holistic approach tackles the problem considering the overall network condition, while the sequential approach is easier to implement and understand, but may lead to solutions far from optimal. Scenarios defining the suitability of these approaches are defined. Finally, an iterative approach gathering the advantages of traditional approaches is proposed and applied in a case study. The proposed approach considers the overall network condition in a simpler and more intuitive manner than the holistic approach.

## 1. Introduction

**Figure 1:**
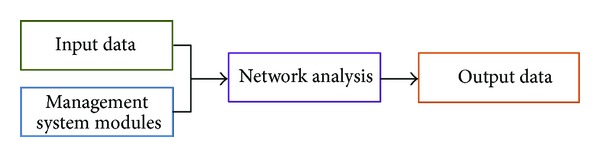
Modules in a PMS used to evaluate the suitability of maintenance programs at the network level.

Pavement management systems (PMS) should assist agencies in the decision making process about which sections of a pavement network should be preserved, maintained, and/or rehabilitated (P + M + R) under budgetary constraints. To address this, a systematic and rational method is needed to ensure an optimal allocation of scarce resources. Studies carried out by the Economic Commission for Latin America and the Caribbean (ECLAC) and the German Agency for Technical Cooperation (GTZ) have shown that between 1% and 3% of gross domestic product is consumed each year unnecessarily due to the lack of road network management [1]. At the same time, users are increasingly demanding in terms of quality, comfort, and safety. Therefore, the design of maintenance programs becomes a crucial aspect when defining the questions about which section to treat, which treatment to apply, and when this treatment must be applied. For this analysis, PMS must integrate three management levels varying in the information detail and complexity of models considered in the decision making: strategic, network, and project level. This study focuses on management at the network level, whose primary purpose is the design of the network maintenance program, within overall budget constraints. In order to evaluate the suitability of maintenance programs at the network level, PMS integrate and coordinate four specific modules (Figure 1) [2].

- *Input Data.* Data required for the network analysis are inventory data per road, network present condition, performance models (including the evolution of pavement condition and the effect of P + M + R treatments), and strategic level data. Strategic level data include strategic targets (i.e., trigger level of service, environmental policies, institutional scopes, and objectives), available budget, analysis period, and discount rate for long term economic analysis.
- *Management System Modules. *This module evaluates the suitability of maintenance alternatives. If a sustainable management is implemented, the evaluation has to consider, in an integrated manner, five aspects throughout the pavement life cycle: economic, social, technical, environmental, and political [3–5]. Different indicators have been used for assessing these sustainable aspects, for example, present worth cost or cost effectiveness as economic indicators, safety and comfort as social indicators, roughness as an indicator of technical performance, air pollution due to vehicle emissions as environmental indicator, and sections' functional class as a political indicator. Once aspects to consider have been selected, managers at the strategic level have to define the decision making criteria, that is, how these sustainable aspects will be considered for evaluating and comparing the suitability of maintenance alternatives.
- *Network Analysis.* Based on information from input data and management system modules, this module seeks the optimal maintenance program at the network level.
- *Output Data.* It mainly consists of the maintenance program at the network level and pavement condition over the analysis period. It could also include recommendations to adjust strategic targets.

Once input data and management system modules are defined, the optimal design of maintenance programs is not straightforward. Indeed, it presents *S* ^*T*×*N*^ possible solutions in a network of *N* sections with *S* possible P + M + R treatments over a planning horizon of *T* years [6]. Given this scenario, two approaches have been identified in the literature to allow the optimization process: sequential and holistic [7, 8]. These approaches differ in how the optimization problem is tackled. Sequential approach deals with the problem in two phases. It first defines the treatment strategy on a section by section analysis. Once the P + M + R treatments and timings are defined for each section, it selects the sections to treat until available budget is exhausted. This sequential approach simplifies the problem by evaluating *N* × *S* ^*T*^ possible solutions. On the other hand, holistic approach tackles the problem as a whole, before any specific section or treatment strategy is defined. Therefore, it deals with the *S* ^*T*×*N*^ solutions of the problem. In addition to these two possible approaches of the problem, different methods can be applied to look for the optimal maintenance program. These optimization methods vary in the number of evaluated alternatives and how the selection is carried out and they can be applied considering either sequential or holistic approach. There is no specific approach and optimization method appropriate to all possible scenarios; therefore, a previous understanding of the problem within an agency is mandatory to identify the most suitable approach for a particular network [8].

Previous works in other research areas (i.e., bridge management and construction engineering) have analyzed the use of existing optimization methods in the decision making process [9, 10]. With respect to pavement management, previous works have analyzed how pavement agencies select the projects to undertake [8, 11]. In 1995, simplistic approaches were detected in most of the American and Canadian agencies. This was mainly due to the lack of computers to undertake more reliable analysis [8]. Recent advances in computer technologies make necessary an update of optimization methods to tackle the problem of designing maintenance programs. A recent study reviews optimization techniques applied to pavement management with special attention to genetic algorithms [11]. However, this review neither compares existing optimization methods nor considers the traditional approaches of the problem (sequential or holistic). Having detected this gap in the literature, the present study aims to assist highway agencies, researchers, and practitioners on when to apply and how to apply available optimization methods for the optimal design of maintenance programs at network level.

The objective of this study is to recommend the most suitable approaches and optimization methods for the design of maintenance programs under different scenarios. Based on an analysis of the current state of the practice, this study proposes an iterative approach that gathers the advantages of traditional approaches (sequential and holistic) by considering the overall network condition in a simpler and more intuitive manner than with a holistic approach.

The study is part of a three-year project developed in Chile by the Pontificia Universidad Católica de Chile (PUC) and named Fondef D09I1018 “Investigación y Desarrollo de Soluciones para la Gestión de Pavimentos Urbanos en Chile” (Research and Development of Solutions for Urban Pavement Management in Chile). The project is being partnered and advised by the Centre for Pavement and Transportation Technology (CPATT) of the University of Waterloo, Canada. The overall project resulted as a cooperative initiative of the PUC and funding partners to accomplish the current and future needs of urban pavements and provide effective management tools to assist agencies that manage urban networks in decision making. Even though the project is being developed in Chile, the expected outcomes, such as technical tools and the resulting Urban Pavement Management System, may be adapted and adopted in other countries for urban pavement management.

## 2. Study Methodology

To achieve the proposed objective, a four-step research method is proposed:

- review of existing optimization methods applied to pavement management including existing applications in available PMS under traditional approaches (sequential and holistic);
- comparative analysis of reviewed approaches and optimization methods, identifying their advantages and limitations; based on this analysis, recommendations of the most suitable approach and optimization method to implement in future PMS are driven considering different scenarios;
- proposal of an iterative approach gathering the advantages of traditional approaches;
- application of the proposed iterative approach in an illustrative case study and comparison to traditional approaches.

## 3. Selection and Optimization of Maintenance Treatments

As shown in Table 1, several optimization methods are available for the design of maintenance programs at the network level. They mainly vary in the number of alternatives considered. Therefore, they are related to the considered approach: sequential approach deals with *N* × *S* ^*T*^ alternatives, while holistic approach considers *S* ^*T*×*N*^ possible solutions (Table 2). Indeed, the suitability of an optimization method relies on the number of alternatives under evaluation: when the number of alternatives is small, they can be selected based on a ranking. In contrast, when the number of alternatives is large, it becomes necessary to use mathematical or near optimization methods. These optimization methods are reviewed in this section including applications in available PMS.

### 3.1. Selection Based on Ranking

Selection based on ranking is performed by enlisting and rating alternatives based on an indicator. This indicator can be based on judgment, pavement condition, or economic analysis.

When based on judgment, agencies determine from an expert panel a criterion to rate and rank alternatives. Shah et al. [12] applied this method to select the sections to treat in a road network in India considering criteria such as traffic, connectivity, and road and drainage conditions.

Selection based on pavement condition ranks sections to treat considering either a Single or a Composite Condition Index. Single Condition Index is normally based on roughness or structural index, whereas Composite Condition Index often considers pavement condition and functional classification. A Composite Condition Index considering pavement surface distresses, traffic information, and expert opinion is used by Reddy and Veeraragavan [13] for selecting sections to treat on a network of 52 sections.

Ranking based on economic analysis allows a rational comparison among alternatives because it considers costs and benefits. This method was used by Shah et al. [12] under a sequential approach. Firstly, for each section, four maintenance strategies were ranked based on a benefit cost ratio. This economic indicator was also used to rank sections to treat. Another application of ranking based on economic analysis has been implemented in MicroPaver PMS using the cost effectiveness for selecting the sections to treat [14].

### 3.2. Mathematical Optimization Methods

Mathematical optimization methods select alternatives maximizing or minimizing an objective function while satisfying some constraints. Objective functions commonly considered are maintenance costs, vehicle operating costs, and effectiveness, among others [2, 6, 15]. Mathematical programming methods most commonly used for pavement management are linear, nonlinear, integer, and dynamic programming. They are discussed in detail below.

Linear and nonlinear programming seek optimal solutions using continuous variables. The main difference is that the former considers linear functions correlated with time, while the latter may consider curvilinear dependency [8]. These optimization methods have been applied using both holistic [16, 17] and sequential approaches [18, 19]. Under the sequential approach, these methods have been used to optimize the treatment strategy at the section level [19] and the sections to treat at the network level [18].

Integer programming simplifies the analysis by considering two variables: a do nothing alternative or to do something. Applications are found using both sequential [20, 21] and holistic approaches [22, 23]. Regarding sequential approach, Ng et al. [20] optimized the treatment strategy at the section level, while Odoki and Kerali [21] implemented this procedure in HDM-4 PMS to select sections to treat at the network level. However, due to the complexity of the calculation, HDM-4 limits this analysis to networks with less than 100 sections, 16 treatments, and five years [21].

Dynamic programming is used in situations that require a number of sequential decisions. This optimization method starts at the desired final solution and works backwards to find the optimal value of variables. Dynamic programming has been applied using holistic [24] and sequential approaches for the treatment strategy optimization [14] and the section selection [25, 26].

### 3.3. Near Optimization or Heuristic Methods

Near optimization methods, also called heuristic methods, give solutions that are close approximations to those derived from mathematical optimization. These optimization methods start with an initial solution and look for better solutions within the constraints. They differ in how they search for better solutions: incremental benefit/cost analysis, local search heuristics, and evolutionary algorithms.

Incremental benefit/cost analyzes the benefits gained by selecting alternatives with higher costs. This optimization method is often referred to as the efficiency frontier. This frontier is defined in a plot of benefit against cost and gathers the alternatives with higher benefits given a certain cost. Incremental benefit/cost analysis is implemented in HDM-4 PMS under a sequential approach for selecting the maintenance strategy and the sections to treat. However, HDM-4 limits this application to a maximum of 400 sections, 17 treatment alternatives, and a 12-year analysis period [21]. Incremental benefit/cost has also been applied to prioritize sections to treat in unpaved networks [2].

Local search heuristics start with random initial solution and explore the solution inference space seeking for better feasible solutions. Different local search heuristic can be implemented: gradient search, threshold acceptance, simulated annealing, and so forth. These heuristics have been applied under a sequential approach to optimize a road section treatment strategy: Tsunokawa et al. [27] looked for the overlay thickness maximizing benefits, while Chou and Le [28] considered two objectives, minimal cost and maximal reliability.

Evolutionary algorithms (EA) mimic the natural evolution guided by learning and adaptation. Among EA, genetic algorithms are one of the most applicable optimization methods in infrastructure management [29]. They have been applied using both sequential (optimal treatment strategy [26] and section selection [30]) and holistic approaches [25, 31, 32].

### 3.4. Other Optimization Methods

This section gathers optimization methods that assist decision making in managing pavement maintenance at the network level but they cannot be categorized in the above groups as neural networks and fuzzy logic.

Neural networks are able to learn from examples, enabling these systems to make generalizations and simulate decisions. Fwa and Chan [33] developed a neural network based on the priority ratings awarded by engineers. After the training phase, it provided rating scores to road sections based on their condition.

Fuzzy logic systems incorporate imprecise qualitative data in the decision making. Moazami et al. [34] applied a fuzzy logic system in a network with 131 sections. In this system, sections were characterized by condition, traffic, road width, and treatment cost. The fuzzy logic system transformed the quantitative values of these parameters into linguistic values that classified priority in three levels: low, medium, or high.

## 4. Comparative Analysis and Recommendations

This section analyzes the advantages and limitations of reviewed optimization methods and recommends the most suitable methods and approaches for future implementation in PMS under various scenarios. Finally, an iterative approach gathering the advantages of sequential and holistic approaches is proposed.

### 4.1. Advantages and Limitations of the Reviewed Optimization Methods

Selection based on ranking is easy to understand but it can only deal with a limited number of alternatives. Ranking based on judgment is the simplest method, but it may be subject to bias and inconsistency resulting in solutions far from optimal [7]. Ranking based on condition is more objective than judgment. However, it does not consider the effect of alternatives on pavement condition over time. Therefore, Amador-Jiménez and Mrawira [18] have defined optimization based on ranking as the least cost effective method. Finally, ranking based on economic analysis allows a rational comparison that includes costs and benefits. Being simple to use, it leads to solutions closer to optimal than other ranking methods [7]. However, it fails to consider externalities that are difficult to monetize.

Mathematical optimization methods provide optimal solutions but they are not suited to deal with large networks. Indeed, mathematical optimization methods cannot handle large number of decision variables because this increases the complexity of the problem and requires long computing time.

In contrast, near optimization or heuristic methods provide simpler and more efficient solutions to large optimization problems. They are suitable to tackle the maintenance management at the network level leading to “good/near optimal” solutions [6]. Heuristic methods, however, should be compared to optimization methods to ensure that they are representing optimal or near optimal solutions [8].

Regarding other optimization methods, neural networks are useful to replicate a pattern and make generalizations. However, they do not guarantee the suitability of the decision taken and they act as a “black box,” being not possible to easily extract the path followed to explain a solution. Finally, fuzzy logic enable introducing rules from experience or intuition but it has no formal algorithms to learn from existing data [29].

### 4.2. Recommendations of Approaches and Optimization Methods

Reviewed applications show a greater reliance on mathematical optimization and near optimization rather than on ranking (Table 1). In addition to this, applications highlight the versatility of the optimization methods for solving the problem. On one hand, an optimization method (dynamic programming, e.g.) can be implemented under a sequential (A7, A8, and A11 in Tables 1 and 2) or a holistic approach (A23 in Tables 1 and 2). On the other hand, different optimization methods can be combined for solving the same problem. For example, Fwa and Farhan [26] (A11 in Tables 1 and 2) used a genetic algorithm for optimizing the treatment strategy and subsequently applied dynamic programming for optimizing section selection, while Feighan et al. [14] (A8 in Tables 1 and 2) combined dynamic programming and selection based on ranking. Therefore, the suitability of an optimization method should be analyzed in conjunction with the approach of the problem.

Sequential approach simplifies the problem making it easier to understand than holistic approach because it defines first the treatment strategy and then selects the sections to treat. Nevertheless, sequential approach ignores the effect on the network as a whole. This may lead to recommending sequential approach for homogeneous or reduced networks in which the overall performance is less compromised by the section by section analysis. Regarding optimization methods, all the analyzed methods (ranking, optimization, and near optimization) have been used under the sequential approach. The recommendation on the optimization method to use would depend on the characteristics of a specific problem. In broad terms, selection based on judgment or condition should be avoided, as they may introduce bias and do not consider the effect of alternatives over time.

Holistic approach enables analyzing network maintenance alternatives as a whole, before any specific treatment strategy or section has been selected. However, this increases the complexity of the problem, making it necessary to use optimization and near optimization methods (Table 1). Reviewed applications using mathematical optimization methods show a trend of limiting the number of variables considered while near optimization methods are able to handle the problem without sacrificing its complexity. For example, the most complex application using optimization considered 6^108^ alternatives ([22], A9 in Tables 1 and 2), while near optimization has optimized a problem with 7^640^ alternatives ([32], A14 in Tables 1 and 2). Therefore, holistic approach using near optimization methods may be recommended to deal with large networks. Nevertheless, results are suggested to be periodically compared to mathematical optimization methods.

### 4.3. Proposed Iterative Approach

**Figure 2:**
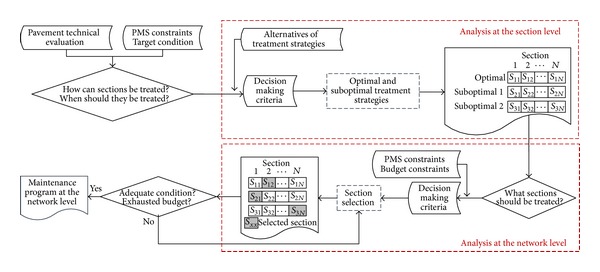
Decision making process of the proposed iterative approach.

Gathering the advantages of sequential and holistic approaches, an iterative approach is proposed as shown in Figure 2. Based on a sequential structure, the iterative approach includes iterations between the selection of treatment strategies and sections looking for a more holistic view of the problem. The proposed approach optimizes first the treatment strategy at the section level. In this optimization, the iterative approach collects a set of optimal and suboptimal solutions. This set of solutions is then considered when selecting the sections to treat at the network level. In this selection, iterations are made looking for the optimal selection of treatment strategies and sections while satisfying budgetary restrictions.

The main difference between sequential and iterative approach is that the latter may select suboptimal treatment strategies for a certain section. On the contrary, reviewed applications considering a sequential approach only consider optimal solutions in the selection of treatment strategies. Therefore, iterative approach enables a deterioration of a solution at the section level if it leads to an improvement of the overall solution at the network level. As a result, the proposed iterative approach considers the overall network condition in a simpler and more intuitively manner than holistic approach.

Several of the reviewed optimization methods (ranking, optimization, and near optimization) may be used considering the proposed iterative approach. As the proposed approach considers two optimizations (treatment strategy and section selection), reviewed optimization methods may be combined. Indeed, iterations are also considered in the reviewed incremental benefit/cost analysis, as shown in the application of Videla and Gaete [35]. The main difference is that incremental benefit/cost analysis only compares solutions in the efficiency frontier. In contrast, the proposed iterative approach may select suboptimal solutions that are not placed in the efficiency frontier. The recommendation of the most suitable optimization method would depend on the number of alternatives to evaluate. If the number of alternatives is reduced, ranking based on economic analysis may be suitable. In contrast, optimization or near optimization methods would be more suitable when dealing with a large number of alternatives. Finally, as the proposed iterative approach considers two optimizations, it enables considering different criteria or objective functions in the selection of treatment strategy and sections to treat. This would therefore facilitate the sustainable management of pavement networks because sustainable aspects, such as economic, social, technical, environmental, and political, may be considered in different optimizations depending on whether they impact at the network or project level.

## 5. Case Study

An illustrative case study is presented to compare the maintenance program obtained under traditional approaches (holistic and sequential) and the proposed iterative approach. The analyzed network, composed of five flexible pavements, is subject to both technical and budgetary restrictions. Each of the sections has a set of six possible rehabilitation treatments and a deterministic deterioration model adopted from Khurshid et al. [36]. Pavement condition is assessed in terms of Present Serviceability Index (PSI) using the regressions proposed by Hall et al. [37]. All the sections present similar characteristics in terms of geometry (1000 m length and 3.7 m width), climate (Average Annual Freeze Index of 400 Celsius days), and traffic (Average Annual Truck Traffic Volume of 0.8 million) while differing on their initial condition (Table 3).

**Figure 3:**
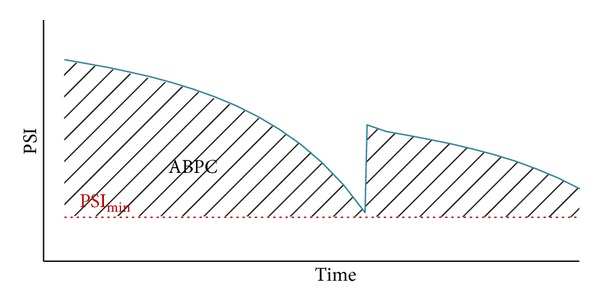
Long term effectiveness of a maintenance alternative.

The maintenance program seeks to maximize long term effectiveness (LTE) over a period of 25 years subject to budgetary restrictions. LTE of maintenance alternatives is assessed by the area bounded by the pavement performance curve (ABPC) and a threshold value (PSI ≥ 2, in this case study) (Figure 3), weighted by traffic and section length [15, 38, 39] (1). The measure of ABPC is based on the fact that a well-maintained pavement (having therefore a larger LTE) provides greater benefits than a poorly maintained pavement [36, 38, 39]. In order to compare alternatives with different costs, the ratio cost effectiveness (*C/E*) (2) is normally considered [15, 39]:

(1) $$\begin{array}{c} \text{LTE}=\text{ABPC}\cdot L\cdot \text{AADT}, \end{array}$$

(2) $$\begin{array}{c} \frac{C}{E}=\frac{\text{LTE}}{\text{TPWC}}, \end{array}$$

where LTE = long term effectiveness, ABPC = area bounded by performance curve and a threshold value (Figure 3),* L* = section length, AADT = annual average daily traffic,* C/E* = cost effectiveness, and TPWC = total present worth cost.

This case study considers an available budget (in terms of total present worth cost, TPWC) 50% higher than the minimal cost solution that ensures a PSI greater than 2. This minimal cost solution (58 220 €) is taken as a base case to compare solutions obtained using different approaches. Although this case study considers an available budget higher than the minimal cost scenario, the proposed approach could deal with lower budgets. In fact, the ultimate goal of the proposed iterative approach is to assist pavement managers on the optimal design of maintenance programs subject to budgetary restrictions. Therefore, other budgetary scenarios could be similarly considered.

A local search heuristic based on simulated annealing was implemented on Matlab 12 in order to look for optimal solutions. Simulated annealing is based on the analogy of crystal formation from masses melted at high temperature and let to cool slowly [40]. This method presents the advantage of escaping from local optima by enabling, under some conditions, the degradation of a solution. This heuristic method, previously implemented by the authors, has led to successful results in large optimization problems [41, 42]. Nevertheless, other optimization methods could be implemented. Indeed, the objective of this application is to analyze the effect of reviewed approaches and not to assess the suitability of the optimization method. The optimization process developed under each approach is the following.

(i) *Holistic Approach*. It consists of the optimization of LTE (1) satisfying both technical and budgetary restrictions (PSI ≥ 2 and TPWC ≤ 87 330 €, resp.).
(ii) *Sequential Approach*. It first optimizes the maintenance strategy at the section level by maximizing the incremental cost effectiveness (IC*/E*, hereafter) (4) while satisfying PSI ≥ 2. Once the optimal maintenance strategy is defined for each pavement, sections with higher IC*/E* (4) are selected until the budget is depleted.
(iii) *Iterative Approach*. it considers the set of three best maintenance strategies for each section based on their incremental cost effectiveness (IC*/E*) (4) assuring that PSI ≥ 2. This set of optimal and suboptimal maintenance strategies is then considered at the network level looking for the maximal LTE (1) while satisfying budget constraint. Therefore, this approach enables the deterioration of a solution at the section level if this would enhance the overall LTE:
  (3) $$\begin{array}{c} \text{IC}=\text{TPWC}-{\text{TPWC}}_{0}, \end{array}$$
  (4) $$\begin{array}{c} \frac{\text{IC}}{E}=\frac{\text{LTE}-{\text{LTE}}_{0}}{\text{TPWC}-{\text{TPWC}}_{0}}, \end{array}$$

where IC = incremental cost of the alternative compared to minimal cost alternative, TPWC = total present worth of the alternative being evaluated, TPWC_0_ = total present worth of minimal cost alternative, LTE = long term effectiveness of alternative being evaluated (assessed by (1)), and LTE_0_ = long term effectiveness of minimal cost alternative (assessed by (1)).

Sequential and iterative approaches tackle the design of maintenance program by optimizing first the incremental cost effectiveness (IC*/E*, (4)) of maintenance strategies for each section in the network (Table 4). The main difference is that, when selecting the sections to treat at the network level, reviewed applications considering a sequential approach only retain optimal maintenance strategies (optimal solutions in Table 4). Meanwhile, iterative approach considers optimal and suboptimal solutions.

**Figure 4:**
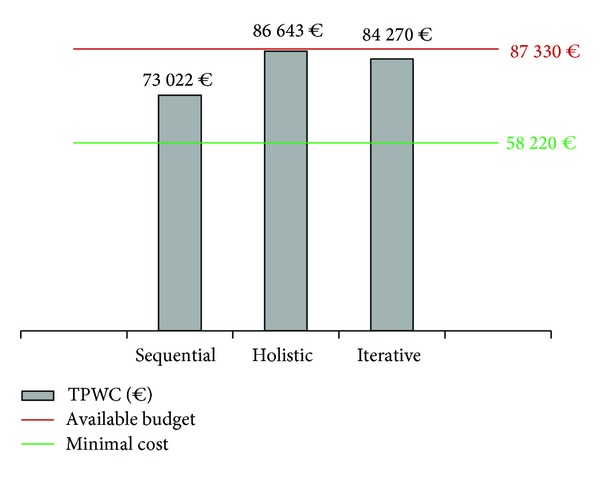
Total present worth of solutions under different approaches.

Considering that the available budget is 50% higher than the minimal cost solution, there is an additional budget of 29 110 €. With this budgetary restriction for improving the network from the minimal cost scenario, sequential approach will solve the optimization problem by only treating Section 2 with the optimal solution (Table 5). It is the first ranked solution in terms of IC/*E* and the cost of the next ranked solution (Section 3: optimal solution) would exceed available budget. However, this solution does not deplete available funds because there is no other optimal solution whose cost does not exceed available budget (Figure 4).

**Figure 5:**
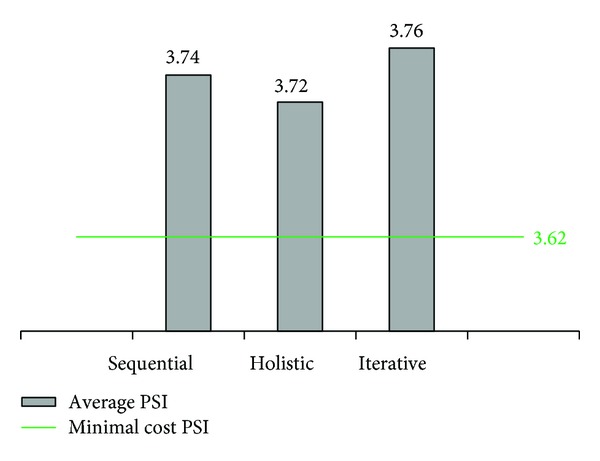
Average PSI of the network under different approaches.

Iterative approach, in contrast, enables the selection of suboptimal solutions at the section level looking for an increase in overall performance (Figure 5). Therefore, iterative approach will treat Section 2 with its optimal solution but it will also treat Section 1 with suboptimal solution 2 (Table 5). This combination results in a total cost closer to the available budget than sequential approach solution (Figure 4). As a result, a higher performance at the network level in terms of average PSI is obtained under iterative approach (Figure 5).

Finally, holistic approach selects a maintenance program based on minimum cost solution except of Section 2, which is treated with a strategy different to those defined as optimal and suboptimal in the section by section analysis. This treatment is referred to as “holistic optimal” (Table 5). In relation to costs and effectiveness, holistic approach nearly depletes available budget (Figure 4) but it does not necessarily ensure an efficient allocation of funds. Indeed, iterative approach leads to a better solution in terms of average PSI with a lower cost than holistic approach (Figures 4 and 5).

From this numerical application it can be concluded that the proposed iterative approach leads to more efficient solutions than sequential approach while considering the overall network condition in a simpler and more intuitive manner than holistic approach.

## 6. Conclusions

From the literature review, two approaches are identified in the design of maintenance programs at the network level: holistic and sequential approach. The former tackles the problem as a whole, before any specific section or treatments are defined, dealing with the *S* ^*T*×*N*^ possible solutions of the problem. Sequential approach tackles the problem considering a two-step process. It first optimizes the maintenance strategy at the section level. Then, budget is allocated across various sections in the network. This process simplifies the problem to *N* × *S* ^*T*^ alternatives.

Different optimization methods can be applied in the design of the maintenance programs at the network level considering either sequential or holistic approach: selection based on ranking, mathematical optimization, near optimization, or heuristic methods and other optimization methods. From the revision of these optimization methods and their applications the following can be concluded.

- Ranking systems are easy to understand, but they can only be used when the number of alternatives is limited and they often ignore future needs.
- Mathematical optimization methods provide optimal solutions, but they require long computing time. They may not be feasible for a large network with long period of analysis.
- Near optimization methods give near optimal solutions with less computational effort than mathematical optimization methods. They can handle large number of decision variables and are suitable to solve combinatorial optimization problems.
- Other optimization methods, such as neural networks and fuzzy logic can replicate a pattern, but they do not guarantee the suitability of the decision taken.

Based on the advantages and limitations of the reviewed optimization methods and their applications under holistic and sequential approaches, several recommendations can be driven for future implementation in PMS.

- Sequential approach is easy to understand but it fails to consider the effect on the network as a whole. It may be recommended for the analysis of homogeneous or reduced networks, in which the overall performance of the network is less compromised by the section by section analysis.
- Holistic approach analyzes network maintenance alternatives as a whole, before any specific treatment strategy or section has been selected. However, this increases the complexity of the problem, making it necessary to use optimization and near optimization methods. Reviewed applications of mathematical optimization methods using holistic approach show a trend of simplifying the problem or limiting the number of variables (sections, treatments, and/or analysis period). Meanwhile, near optimization methods are able to solve the problem under a holistic approach with no sacrificing of its complexity. Therefore, holistic approach using near optimization methods may be recommended when dealing with large networks.

Finally, an iterative approach is proposed and applied to an illustrative case study. This approach gathers the advantages of sequential and holistic approaches leading to more intuitive and effective design of maintenance programs at the network level. Based on a sequential structure, the proposed iterative approach includes iterations between the selection of treatment strategies and sections to treat looking for a more holistic view of the problem. In this iteration process, the proposed iterative approach may select suboptimal treatment strategies for a certain section if it leads to an improvement of the overall solution at the network level. As a result, the proposed iterative approach considers the overall network condition in a simpler and more intuitive manner than holistic approach.

## Figures and Tables

**Table 1:** Reviewed optimization methods consider either sequential or holistic approach.

		Sequential approach		Holistic approach
Optimization method		Treatment strategy	Section selection
Selection based on ranking	Judgment		A20	
	Pavement condition		A19	
	Economic analysis	A20	A8, A20	

Mathematical optimization methods	Linear and nonlinear programming	A12	A1	A6, A13
	Integer programming	A16	A17	A9, A22
	Dynamic programming	A8	A7, A11	A23

Near optimization or heuristic methods	Incremental benefit/cost analysis	A18	A2, A18	
	Local search heuristics	A5, A21		
	Evolutionary algorithms	A11	A3, A7	A4, A14

Other optimization methods	Neural networks		A10	
	Fuzzy logic		A15	

*Note.* Code reference (A1, A2,…, A23) is defined in Table 2.

**Table 2:** Number of alternatives and type of approach considered in reviewed applications.

Code	Author		Problem				Approach		Reference
			*N*	*S*	*T*	Alternatives	Sequential	Holistic	
A1	Amador-Jiménez and Mrawira		3	—	30	3^30^	x		[18]
A2	Chamorro		39	4	10	39 × 4^10^	x		[2]
A3	Chan et al.		500	—	—	500	x		[30]
A4	Chootinan et al.		35	4	10	4^35×10^		x	[31]
A5	Chou and Le		1	15	15	15^15^	x		[28]
A6	De La Garza et al.		5	9	15	9^5×15^		x	[16]
A7	Farhan and Fwa		150	4	1	150 × 4^1^	x		[25]
A8	Feighan et al.		14	5	5–15	14 × 5^15^	x		[14]
A9	Ferreira et al.		27	6	4	6^27×4^		x	[22]
A10	Fwa and Chan		128	—	—	128	x		[33]
A11	Fwa and Farhan		150	4	1	150 × 4^1^	x		[26]
A12	Gao and Zhang		—	4	5	4^5^	x		[19]
A13	Gao et al.		3	4	10	4^3×10^		x	[17]
A14	Meneses and Ferreira		32	7	20	7^32×20^		x	[32]
A15	Moazami et al.		131	—	—	131	x		[34]
A16	Ng et al.		—	4	5–10	4^10^	x		[20]
A17	Odoki and Kerali	Integer program.	100	16	5	x	x		[21]
A18		Increm. benefit cost	400	17	12	x	x		[21]
A19	Reddy and Veeraragavan		52	—	—	52	x		[13]
A20	Shah et al.		21	4	10	21 × 4^10^	x		[12]
A21	Tsunokawa et al.		—	5	20	5^20^	x		[27]
A22	Wang et al.		10	5	5	5^10×5^		x	[23]
A23	Yoo and Garcia-Diaz		40	4	7	4^40×7^		x	[24]

**Table 3:** Characteristics of sections considered in the case study.

Section	Type	Time since last rehabilitation (years)
1	Minimal SP with 102 mm ACO	15
2	Minimal SP with saw and seal 102 mm ACO	20
3	Intensive SP with 102 mm ACO	20
4	Crack break and seat section with 102 mm ACO	25
5	Crack break and seat section with 203 mm ACO	25

*Note.* SP: surface preparation; ACO: asphalt concrete overlay.

**Table 4:** Optimal and suboptimal treatment strategies considered in the iterative approach.

Treatment strategy solution	Section 1		Section 2		Section 3		Section 4		Section 5	
	IC (€)	IC/*E *	IC (€)	IC/*E *	IC (€)	IC/*E *	IC (€)	IC/*E *	IC (€)	IC/*E *
Optimal	40 773	0.88	14 802	3.84	20 343	1.47	43 183	0.94	19 970	1.18
Suboptimal 1	53 220	0.71	33 251	2.38	43 470	1.36	55 349	0.73	26 725	0.88
Suboptimal 2	11 248	0.67	32 136	2.29	54 550	1.10	68 002	0.60	39 339	0.60

**Table 5:** Treatment strategies for the different sections of the network under different approaches.

	Sequential	Holistic	Iterative
Section 1	MC	MC	Suboptimal 2
Section 2	Optimal	Holistic optimal	Optimal
Section 3	MC	MC	MC
Section 4	MC	MC	MC
Section 5	MC	MC	MC

*Note.* MC corresponds to minimal cost treatment strategy.
